# Ascorbic Acid Reduces the Blood Boss After Total Knee Arthroplasty: Insights From a Randomized Controlled Trial

**DOI:** 10.1016/j.artd.2025.101618

**Published:** 2025-02-01

**Authors:** Pooya Hosseini-Monfared, Alireza Mirahmadi, Mohammad Mehdi Sarzaeem, Soheil Pourshahryari, Parisa Aminnia, Mohammad Poursalehian, Seyed Morteza Kazemi

**Affiliations:** a Bone Joint and Related Tissues Research Center, Shahid Beheshti University of Medical Sciences, Tehran, Iran; b Imam Hossein Hospital, Shahid Beheshti University of Medical Sciences, Tehran, Iran; c Joint Reconstruction Research Center, Tehran University of Medical Sciences, Tehran, Iran

**Keywords:** Blood loss, Antioxidants, Total knee arthroplasty, Ascorbic acid

## Abstract

**Background:**

Blood loss is among the main complications of total knee arthroplasty (TKA) and oxidative stress, and hemolysis caused by reactive oxygen species are one of the causes of hemoglobin (Hb) drop. Ascorbic acid is a potent antioxidant that can protect against reactive oxygen species. In this study, we aim to explore the antioxidant effect of ascorbic acid on blood loss and patient-reported outcomes following outpatient TKA.

**Methods:**

Patients scheduled for outpatient primary TKA were enrolled in this randomized, double-blind clinical trial and were assigned to 1 of the 2 groups. The patients in the ascorbic acid group received intravenous vitamin C perioperatively. Patients in the placebo group received only normal saline. We calculated the blood loss using the Hb drop. Patient-reported outcomes such as Oxford Knee Score, Western Ontario and McMaster Universities Osteoarthritis Index, Knee Injury and Osteoarthritis Outcome Score, and Forgotten Joint Score were used to evaluate the postoperative pain and function in the 6-month follow-up.

**Results:**

The patients who have received ascorbic acid had lower Hb drop (g/dL) (1.30 ± 0.72 vs 1.91 ± 0.84, *P* value < .001) and total blood loss in the first postoperative day (463.60 ± 274.37 vs 732.11 ± 347.78, *P* value < .001). Also, fewer patients reached the minimum clinically important difference level for Hb drop in the ascorbic acid group. The patients’ postoperative functional and pain scores were not different between the 2 groups.

**Conclusions:**

Our findings demonstrated that perioperative use of ascorbic acid can reduce blood loss by nearly 36% on the first postoperative day and should be considered as an effective blood-preserving agent in conjunction with tranexamic acid during TKA.

## Introduction

Total knee arthroplasty (TKA) is a highly effective surgical intervention for individuals suffering from end-stage osteoarthritis [1]. Blood loss following TKA is one of the complications of TKA, potentially leading to transfusion-related allergic reactions, blood-borne infections, periprosthetic infections, longer hospital stays, and higher morbidity [2,3]. Minimizing blood loss is an important objective to enhance patient outcomes and optimize the healthcare resources associated with TKA.

Total blood loss (TBL) estimated by hemoglobin (Hb) drop after the surgery is usually more than the visible blood loss during the surgery and the difference between the TBL and visible blood loss is called hidden blood loss (HBL) [4]. Several studies have investigated the pathological mechanism of HBL in TKA. It has been demonstrated that insertion of the femur and tibia intramedullary guides during TKA may trigger oxidative stress through reactive oxygen species (ROS) [5]. The ROS reacts with polyunsaturated fatty acids, disrupts the membranes of the red blood cells (RBCs), and causes hemolysis, resulting in Hb drops and HBL after the procedure [6,7]. Identifying the aforementioned mechanisms raises the possibility that antioxidants may be effective agents to reduce blood loss following TKA.

Ascorbic acid (vitamin C) is a water-soluble and widely used antioxidant that has been proven to protect cells against ROS [8]. The ascorbic acid’s effect on blood loss has been studied in nonorthopaedic surgeries, and it has been proved to be effective in reducing the blood loss after these surgeries [[9], [10], [11]]. It is believed that in addition to the anti-inflammatory and antioxidant effect, ascorbic acid’s role in platelet aggregation is the underlying mechanism of this positive effect [12]. Ascorbic acid has been shown to stabilize the endothelium and reduce endothelial permeability, which could theoretically reduce the incidence of perioperative bleeding [13]. Ascorbic acid depletion has also been established in patients who underwent TKA, indicating significant oxidative stress during TKA [14,15].

Despite these theoretical benefits, clinical evidence supporting the efficacy of perioperative intravenous (IV) ascorbic acid in reducing blood loss during TKA is lacking. Our study aimed to address the gap in the literature by a randomized clinical trial to investigate the effect of IV perioperative administration of ascorbic acid on blood loss in patients undergoing outpatient TKA compared with the control group without receiving ascorbic acid. Our study is designed to answer 2 pivotal questions: (1) Can ascorbic acid reduce perioperative blood loss following TKA? (2) Can ascorbic acid improve patient-reported outcome measures following TKA?

## Material and methods

### Study design

In this triple-blinded, randomized, controlled clinical trial, we evaluated patients undergoing primary TKA between June and August 2023 at our institution in an outpatient setting. Informed consent was obtained from all the patients prior to the surgery. The study received ethical approval from the Institutional Review Board (IR.SBMU.RETECH.REC.1402.165), ensuring compliance with ethical guidelines and transparency in the research process and was registered under the clinical trial registry (IRCT20221104056393N1).

### Eligibility criteria

Inclusion criteria were considered as patients with knee pain who were diagnosed with osteoarthritis and were candidates for primary TKA. Exclusion criteria were as follows:

Patients with a preoperative Hb level below 10 g/dL, individuals with clotting disorders, as indicated by abnormal prothrombin time, partial thromboplastin time, or international normalized ratio, women in their menstrual period, pregnant or breastfeeding individuals, patients with severe renal dysfunction or severe infections, individuals with a history of thromboembolic events, patients diagnosed with inflammatory arthritis, such as rheumatoid arthritis or pigmented villonodular synovitis, substance abusers, including drugs and alcohol, patients with conditions contraindicating the use of vitamin C, such as glucose-6-phosphate dehydrogenase deficiency, sickle cell disease, and hemochromatosis, and patients currently taking medications that interact with vitamin C, including chemotherapy drugs [[16], [17], [18]].

### Allocation and blinding

Patients were given a study identification number and assigned to the ascorbic acid or control group using a computerized random number generator. The patients, research fellows, the surgeon, and the person who analyzed the data were blinded to the patient’s group. The intervention agent (ascorbic acid) and placebo (saline) were sealed in a similar package to blind the patients and the surgeon.

### Surgical technique and postoperative protocol

Patients received standard spinal anesthesia using 25-50 μg of fentanyl and ropivacaine 0.5% (Molteni & C Dei Fratelli, Italy). Intraoperative sedation was provided based on the anesthesiologist’s discretion using a 7-10 mL/h infusion of propofol 2% (Fresenius Kabi Austria Gmbh, Austria).

A single experienced surgeon performed all TKAs using an anterior midline skin incision and the subvastus approach [19]. All participants received cruciate-retaining Zimmer Persona (Zimmer, Warsaw, Indiana) prostheses. A pneumatic tourniquet was employed for all patients with a pressure set approximately 100 mmHg above the systolic blood pressure. The tourniquet was inflated before the incision and deflated after the skin was closed. We did not use vacuum drains or blood salvage systems. Tranexamic acid (TXA) with the dose of 1 g was injected intravenously prior to the tourniquet application and also 1 g of TXA was injected intra-articularly during the final stage of the TKA.

Patients in the ascorbic acid group were administered the ascorbic acid following the method outlined in similar previous studies [9]. The first ascorbic acid dose (1 g in 10 mL) was given via IV at the beginning of the surgery. The second dose of ascorbic acid (1 g in 10 mL) was infused after the tourniquet release. After the operation, the patients received an additional dose of ascorbic acid (1 g in 10 mL) during the first 6 hours after TKA. In the control group, patients received an equivalent volume of saline as a placebo at the same frequency.

Following TKA, patients were dressed with an elastic bandage and a knee immobilizer. As part of the postoperative care, enoxaparin 40 mg was administered once daily subcutaneously, starting 12 hours after the surgery and continued for 14 days. Cefazolin was used as antimicrobial prophylaxis, administered within 60 minutes prior to the surgical incision, and repeated every 8 hours up to 24 hours after the surgery. Patients followed similar postoperative rehabilitation protocol including lower extremity muscle strength training and walking exercise.

Blood transfusions were considered for patients with Hb levels below 7 g/dL or below 8 g/dL if they exhibited clinical symptoms of anemia, such as lightheadedness, fatigue, palpitations, or shortness of breath [20,21].

### Variables and outcome measures

Patients’ demographics, such as gender, body mass index, age, and preoperative Hb level were recorded.

#### Primary outcome

The primary outcome measure was the amount of HBL. We employed the Hb balance method, which takes into account the postoperative drop in Hb level 24 hours after surgery and estimated blood volume adjusted for the weight and height of the patients using the formula developed by Nadler et al. to determine the TBL [[22], [23], [24]]. To rule out dilutional Hb drop, the patients were clinically examined to be in the euvolemic state for postoperative Hb measurements. Due to using a tourniquet during these surgeries, we observed minimal and negligible visible intraoperative blood loss. Furthermore, there was no apparent postoperative blood loss in the form of hematoma or swelling, assessed by clinical examination. In cases of suspicious swelling, an ultrasound of the knee was used [25]. Therefore, all measured blood loss, as indicated by the drop in Hb levels, can be attributed to HBL.

#### Secondary outcomes

The blood transfusion rate at both intraoperative and postoperative states was recorded. We incorporated the visual analog scale for pain, Oxford Knee Score, Western Ontario and McMaster Universities Osteoarthritis Index, Knee Injury and Osteoarthritis Outcome Score, and Forgotten Joint Score preoperatively and at 6 months of follow-up [[26], [27], [28], [29]].

Incidence of thromboembolic events, infection, failure of the implant, need for reoperation, incidence of effusion and edema, stiffness, and wound complications were also evaluated at the final follow-up.

### Sample size

The primary outcome measure was the amount of blood loss after TKA. A power analysis using a 2-tailed, 2-sample *t*-test determined that a sample size of 52 patients in each group was satisfactory to detect 25 mL change of TBL, with an assumed alpha value of 0.05 and power of 80% [30]. We did not consider a drop-out rate for this study since all patients were available on postoperative day 0-1.

### Data analysis

Statistical analysis was executed using SPSS statistical software version 29 (IBM, Armonk, New York) and GraphPad Prism version 9.5.1 (GraphPad Prism Software Inc., Boston, MA, USA). To analyze continuous variables that were normally distributed, we used the Student’s *t*-test and 1-way analysis of variance. In addition, we used the Mann-Whitney *U* test and Kruskal-Wallis test for analyzing continuous variables that were not normally distributed. The Pearson Chi-squared test was used for categorical variables analysis. Regression models were made to evaluate independent prediction factors related to Hb drop as a dependent factor. Receiver operating characteristic (ROC) curve analysis was used to determine the cut-off for preoperative Hb to predict postoperative anemia. We considered *P* values less than .05 to be statistically significant.

## Results

**Figure 1:**
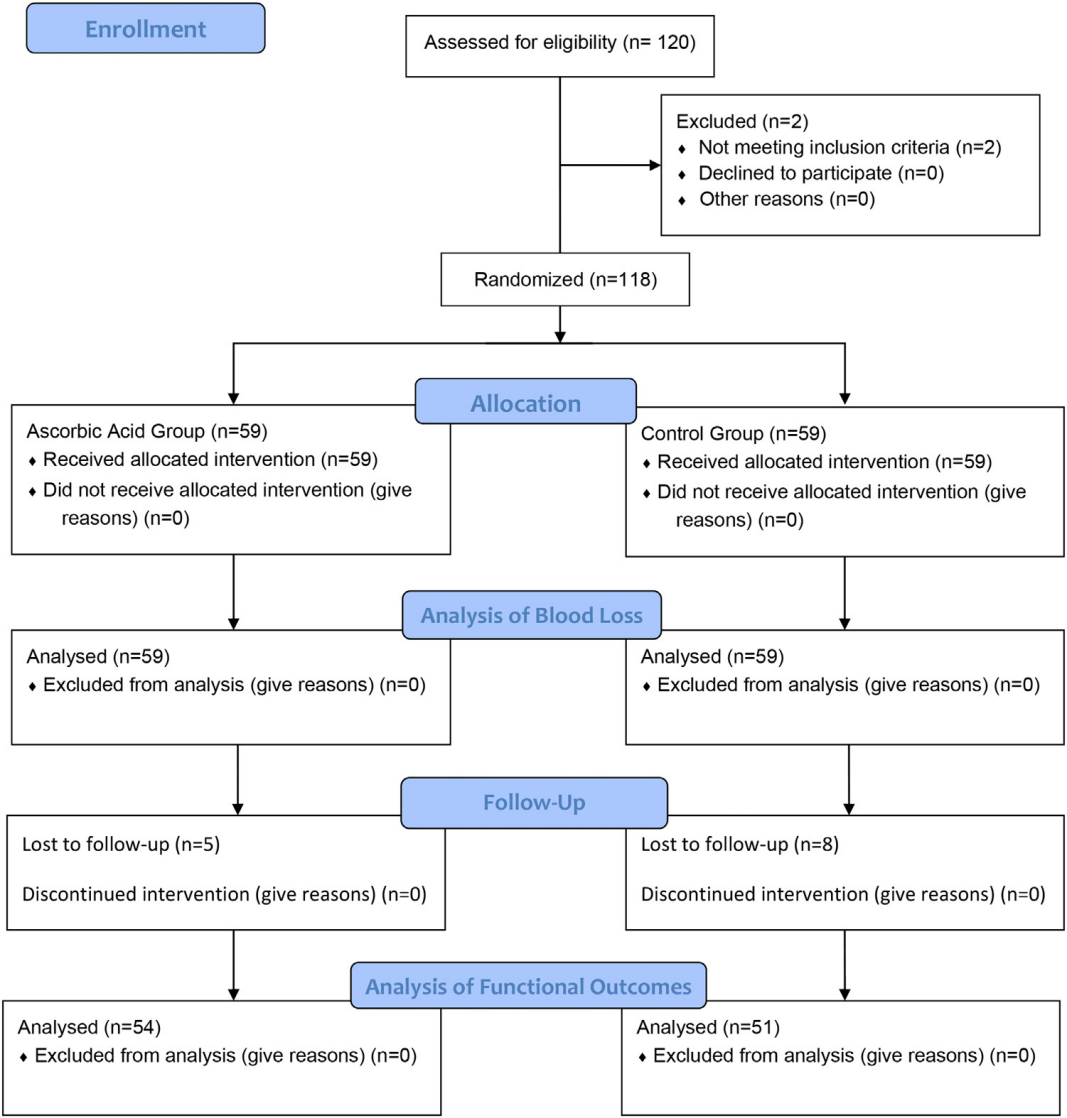
CONSORT flow diagram of the study. CONSORT, Consolidated Standards of Reporting Trials.

After considering the exclusion criteria, 118 patients were included in this study. The recruitment process and progression of the study are presented in the Consolidated Standards of Reporting Trials flow diagram template in Figure 1.

The patients had a median age of 69 years (range: 51-89). Women formed 83.9% (n = 99) of the patients. The 2 groups’ demographic features, preoperative Hb level, and functional scores did not show any statistically significant difference (Table 1).

**Table 1:** The demographic characteristics of the included patients (mean ± SD).

Variable	Total, n = 118 (100%)	Ascorbic acid group, n = 59 (50%)	Control group, n = 59 (50%)	*P* value
Gender				
Men	19 (16.10)	9 (15.25)	10 (16.95)	.802
Women	99 (83.90)	50 (84.75)	49 (83.05)	
Age (y)	69.42 ± 6.37	68.61 ± 6.83	70.24 ± 5.81	.195
BMI (kg/m^2^)	28.40 ± 3.89	28.82 ± 3.77	27.97 ± 3.99	.145
Healthy weight (18.5-25)	19 (16.1)	8 (13.6)	11 (18.6)	.420
Overweight (25-30)	67 (56.8)	32 (54.2)	35 (59.3)	
Obesity (>30)	32 (27.1)	19 (32.2)	13 (22.0)	
Blood volume (L)	4.13 ± 0.51	4.08 ± 0.48	4.18 ± 0.52	.282
Preoperative Hb level (g/dL)	13.06 ± 1.35	13.00 ± 1.29	13.13 ± 1.42	.623
Preoperative VAS	8.65 ± 1.48	8.42 ± 1.54	8.84 ± 1.42	.212
Preoperative WOMAC	28.59 ± 22.89	23.48 ± 15.89	33.70 ± 27.59	.260
Preoperative OKS	44.87 ± 10.16	45.48 ± 11.18	44.45 ± 9.49	.454
Preoperative FJS	72.46 ± 22.45	77.59 ± 15.01	67.33 ± 27.34	.275
Preoperative KOOS				
Pain	39.33 ± 3.70	39.16 ± 3.82	39.46 ± 3.62	.690
Symptoms	38.04 ± 3.42	37.97 ± 3.21	38.09 ± 3.61	.876
ADL	42.25 ± 6.56	42.34 ± 6.21	42.16 ± 6.88	.897
Sport/recreation	17.19 ± 3.87	17.04 ± 3.84	17.31 ± 3.92	.606
Quality of life	25.27 ± 4.23	25.05 ± 4.18	25.44 ± 4.30	.663

ADL, activities of daily living; BMI, body mass index; FJS, Forgotten Joint Score; Hb, hemoglobin; KOOS, Knee Injury and Osteoarthritis Outcome Score; OKS, Oxford Knee Score; SD, standard deviation; VAS, visual analog scale; WOMAC, Western Ontario and McMaster Universities Osteoarthritis Index.

We found that postoperative Hb drop was lower in the ascorbic acid group (1.30 ± 0.72 vs 1.91 ± 0.84, *P* value < .001), with the Cohen’s effect size of 0.78 indicating large effect size.

Our findings demonstrated that TBL (mL) calculated by the Hb balance formula was lower in the ascorbic acid group (463.60 ± 274.37 vs 732.11 ± 347.78, *P* value < .001). None of the patients in our study required a blood transfusion, as the lowest postoperative Hb level recorded was 8.2 g/dL, which was above the predefined cut-off value for blood transfusion (Table 2).

**Table 2:** Early postoperative outcomes of the study groups (mean ± SD).

Variable	Ascorbic acid, n = 59	Control, n = 59	95% confidence intervals	*P* value
Postoperative Hb level (g/dL)	11.69 ± 1.20	11.21 ± 1.20	0.046-0.926	.031^a^
Hb drop (g/dL)	1.30 ± 0.72	1.91 ± 0.84	0.324-0.896	<.001^b^
Total blood loss (mL)	463.60 ± 274.37	732.11 ± 347.78	154.28-382.73	.000^b^
Transfusion rate	0 (0%)	0 (0%)		

Hb, hemoglobin; SD, standard deviation.

a *P* value < .05.

b *P* value < .001.

According to previous studies, a Hb change of 2 g/dL was considered a clinical important change of Hb [31,32]. Fewer number of patients who received ascorbic acid reached the minimum clinically important difference for Hb drop than patients in the control group (15.3% compared to 52.5%, *P* value < .001).

**Figure 2:**
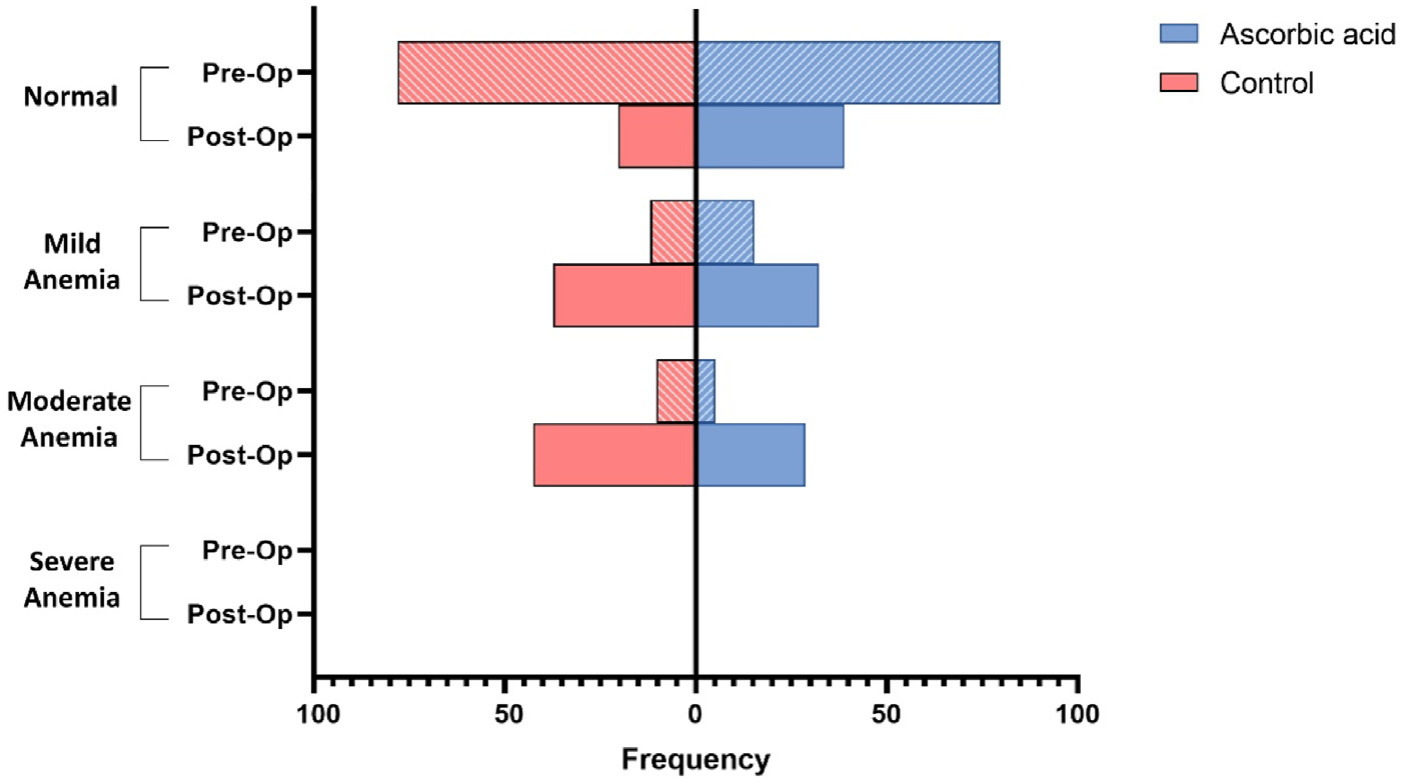
The comparison of anemia progression in the preoperative and postoperative stages between the 2 study groups.

In total, 21.2% (13.6% mild and 7.6% moderate) of the individuals included in our study had anemia in the preoperative stage, according to the World Health Organization criteria [33]. Following TKA, 70.3% (34.7% mild and 35.6% moderate) of the patients had postoperative anemia. The progression of anemia in the 2 study groups has been demonstrated in Figure 2.

As ascorbic acid reduced Hb drop significantly, the incidence of anemia was higher in the control group (*P* = .039) (Table 3). Despite the increased incidence of anemia, there was no significant correlation between postoperative functional and pain scores and the existence of preoperative and postoperative anemia (*P* > .05).

**Table 3:** Preoperative and postoperative incidence of anemia.

Variable	Ascorbic acid, n = 59	Control, n = 59	*P* value
Preoperative			.822
Normal	47 (79.7%)	46 (78%)	
Anemia	12 (20.3%)	13 (22%)	
Postoperative			.027^a^
Normal	23 (39%)	12 (20.3%)	
Anemia	36 (61%)	47 (79.7%)	

a *P* value < .05.

Multivariate logistic regression was conducted to establish the effects of age, gender, body mass index, preoperative Hb, and the use of ascorbic acid on postoperative anemia. The model explained 62.7% (R^2^) of the variance of postoperative anemia and correctly classified 83.9% of the cases. Gender (*P* = .003, B = −3.468, odds ratio [OR] = 0.031, 95% confidence interval [CI] = 0.003-0.297), the use of ascorbic acid (*P* = .002, B = 2.157, OR = 8.643, 95% CI = 2.193-34.063), and preoperative Hb (*P* < .001, B = −2.099, OR = 0.123, 95% CI = 0.049-0.304) had a significant effect on the development of postoperative anemia. Overall, being male, having lower preoperative Hb, and not using ascorbic acid was associated with an increased likelihood of postoperative anemia.

**Figure 3:**
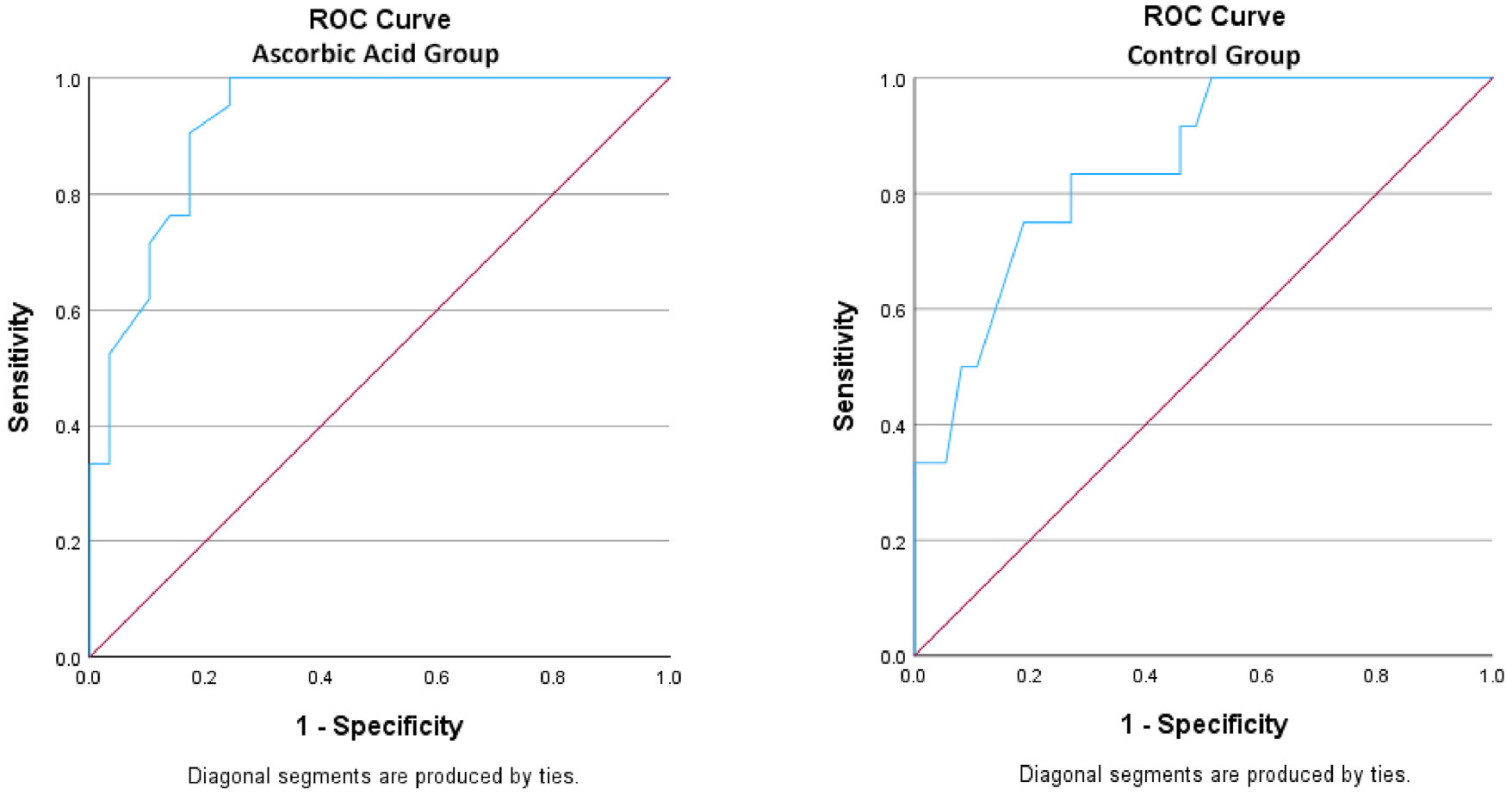
ROC curves of ascorbic acid and control groups for determining the cut-off value for postoperative anemia in women. ROC, receiver operating characteristic.

To compare the preoperative Hb level cut-off values for postoperative anemia between the ascorbic acid and the control group, an ROC curve analysis was conducted. The results revealed that for women, the administration of ascorbic acid reduced the minimum preoperative Hb level cut-off value from 13.95 to 12.75 (Fig. 3) (Table 4). As all the men in the control group had postoperative anemia, ROC curve analysis for this group was not possible. Although for men in the ascorbic acid group, the analysis showed preoperative Hb 14.60 as the cut-off for predicting postoperative anemia (*P* = .040, area under the curve = 100%, Youden’s index = 100%, and sensitivity and specificity = 100%). Due to the low number of men in both the control (with and without postoperative anemia were 10 vs 0, respectively) and ascorbic acid groups (with and without postoperative anemia were 7 vs 2, respectively), the result of ROC analysis for this group could increase bias in the study, so it was not mentioned in Table 4.

**Table 4:** ROC curve analysis for determining the cut-off value for postoperative anemia in women.

	Preoperative Hb cut-off value	Sensitivity (%)	Specificity (%)	Youden’s index (%)	AUC (%)	*P* value
Ascorbic acid	12.75	100	75.9	75	92.5	.000^a^
Control^b^	13.95	75	81.1	56	84.9	.000^a^
	13.65	83.3	73	56	84.9	.000^a^

AUC, area under the curve; Hb, hemoglobin; ROC, receiver operating characteristic.

a *P* value < .001.

b Two preoperative Hb cut-off values with similar highest Youden’s index were chosen for the control group.

The functional outcome of the patients was evaluated at the 6-month follow-up. Five patients of the ascorbic acid group and 8 patients from the control group were lost to follow-up. According to the postoperative functional scores, patients who received ascorbic acid exhibited better functional scores compared to the control group, but the differences were not statistically significant (*P* > .05). The detailed findings are demonstrated in Table 5.

**Table 5:** Postoperative functional scores of the patients (mean ± SD).

Variable	Ascorbic acid group, n = 54	Control group, n = 51	*P* value
WOMAC	15.25 ± 9.24	21.30 ± 16.63	.284
OKS	39.26 ± 4.62	36.24 ± 7.74	.189
FJS	61.11 ± 12.59	51.69 ± 17.58	.097
KOOS			
Pain	87.22 ± 8.43	84.46 ± 17.26	.629
Symptoms	89.76 ± 9.17	89.81 ± 9.89	.936
ADL	84.41 ± 9.54	76.90 ± 17.72	.224
Sport/recreation	48.50 ± 28.31	36.11 ± 24.46	.084
Quality of life	91.87 ± 10.90	88.88 ± 17.95	.761

ADL, activities of daily living; FJS, Forgotten Joint Score; KOOS, Knee Injury and Osteoarthritis Outcome Score; OKS, Oxford Knee Score; SD, standard deviation; WOMAC, Western Ontario and McMaster Universities Osteoarthritis Index.

No signs of thromboembolic events, infection, failure of the implant, need for reoperation, incidence of effusion and edema, stiffness, hematoma, and wound complications were detected in the follow-up period.

## Discussion

Blood loss following TKA is an important complication. The results of our study demonstrated that patients who received TXA and ascorbic acid had lower amounts of TBL compared to those who received only TXA. The ascorbic acid reduced the TBL by around 36% in the first postoperative day. Our findings also show that receiving ascorbic acid and a higher preoperative level of Hb are associated with less postoperative anemia in the patients after TKA.

It is suggested that fatty emboli and consequent free fatty acids (FFAs) are responsible for blood loss after arthroplasty surgeries [6]. These fat emboli, released by medullary canal instrumentation, metabolize to FFAs that stimulate neutrophils to produce ROS. The oxidation effect of ROS on the RBCs and Hb results in Hb drop and HBL [6,34,35]. In 2022, Yuan et al. investigated the pathological mechanism of HBL by measuring the changes of FFA, ROS, Hb, and RBCs in patients who underwent TKA. They found that following TKA, Hb and RBC decreased significantly, while FFA and ROS levels were substantially elevated. Also, a microscope analysis was performed to examine the morphologic changes of blood cells, and the damaged RBCs were observed. They concluded that oxidative stress and damage to the RBCs were the underlying mechanisms of HBL after the TKA [7].

Several studies of patients who underwent orthopaedic surgeries such as TKA and total hip arthroplasty showed that the plasma level of ascorbic acid is reduced after the surgery [14,[36], [37], [38]]. This finding demonstrated that ascorbic acid is consumed in response to increased levels of ROS, oxidative stress, and inflammation. It has been shown that vitamin C administration reduces postoperative inflammation markers following TKA [39]. Also, the depletion of vitamin C due to oxidative agents released after the surgery is observed in other surgical fields, such as general surgery and cardiovascular surgery [15].

Ascorbic acid has been demonstrated to be effective in reducing blood loss, surgery duration, and hospitalization length in other nonorthopaedic surgeries like abdominal myomectomy (laparotomy), hysterectomy, and functional endoscopic sinus surgery [9,10,40]. It has been suggested that the ascorbic acid’s mechanism of action in surgeries is due to improving platelet aggregation and prevention of platelet depletion during the hemostasis process [12]. In a randomized controlled trial evaluating the effect of TXA on the amount of blood loss following total hip arthroplasty, tablets of vitamin C (1 g) were used as placebo [41]. They found that patients who received only TXA had lower blood loss compared to the placebo group. In this study, we compared the effect of combined TXA and IV ascorbic acid with TXA only group and found that patients who received both TXA and ascorbic acid had less amount of blood loss following TKA.

Studies investigating the effect of vitamin C supplementation on the pain, functional outcomes, and arthrofibrosis after orthopaedic procedures revealed that vitamin C reduced the risk of complex regional pain syndrome (CRPS) but not effective regarding the risk of arthrofibrosis and functional outcomes in patients [14,42,43]. Also, meta-analyses have shown that ascorbic acid is effective in preventing CRPS following distal radius fractures [44,45]. Our results demonstrated that the functional scores of the patients were not significantly different between the 2 groups. Also, the pain level determined by visual analog scale and pain subscores of Western Ontario and McMaster Universities Osteoarthritis Index, Oxford Knee Score, and Knee Injury and Osteoarthritis Outcome Score was not significantly different in patients who received vitamin C compared to the control group. The observed results in our study might be attributed to the short-term administration of vitamin C, as ascorbic acid plasma levels return to normal after approximately 16 hours. It is worth noting that different questionnaires were used in the 2 previous studies to evaluate overall postoperative pain and CRPS.

Our study is subject to several limitations worth mentioning. First, our assessment was confined to patients undergoing TKA in an outpatient setting. A significant limitation was our inability to measure Hb levels on postoperative day 3 and day 5. This limitation arose because our institutional review board declined to approve additional postsurgery laboratory tests [46]. Additionally, the socio-economic constraints of our setting prevented us from evaluating plasma levels of ascorbic acid both before and after the surgery. The cost of measuring vitamin C levels was expensive. Moreover, we did not explore the impact of varying doses of ascorbic acid, which may have the potential to enhance the clinical benefits of this intervention. Another limitation was the loss of some participants during follow-up, potentially affecting the results of patient-reported outcome measures. Despite these constraints, our findings suggest that vitamin C, even when used in conjunction with TXA, can reduce blood loss.

## Conclusions

This study demonstrated that administration of ascorbic acid can reduce TBL by nearly 36% in the first postoperative day following TKA. Our findings also show that receiving ascorbic acid and a higher preoperative level of Hb are associated with less postoperative anemia after TKA. The functional outcomes of the patients who received ascorbic acid were similar to the control group at the 6-month follow-up.

## Availability of data and materials

The data used and analyzed during the present study are available from the corresponding author on reasonable request.

## Consent for publication

Consent forms for publication were obtained from every patient is this study.

## Ethics approval and consent to participate

The study received ethical approval from the Institutional Review Board (IR.SBMU.RETECH.REC.1402.165) and was duly registered in the Iranian Registry of Clinical Trials (IRCT20221104056393N1), ensuring compliance with ethical guidelines and transparency in the research process.

## Conflicts of interest

The authors declare there are no conflicts of interest.

For full disclosure statements refer to https://doi.org/10.1016/j.artd.2025.101618.

## CRediT authorship contribution statement

**Pooya Hosseini-Monfared:** Writing – original draft, Supervision, Project administration, Methodology, Formal analysis, Data curation, Conceptualization. **Alireza Mirahmadi:** Writing – original draft, Methodology, Formal analysis, Conceptualization. **Mohammad Mehdi Sarzaeem:** Writing – review & editing, Investigation, Data curation, Conceptualization. **Soheil Pourshahryari:** Writing – original draft, Investigation, Data curation. **Parisa Aminnia:** Writing – original draft, Investigation, Data curation. **Mohammad Poursalehian:** Writing – review & editing, Methodology. **Seyed Morteza Kazemi:** Writing – review & editing, Supervision, Resources, Project administration, Methodology, Conceptualization.

## Appendix A Supplementary data

Conflict of Interest Statement for Mirahmadi

Conflict of Interest Statement for Sarzaeem

Conflict of Interest Statement for Poursalehian

Conflict of Interest Statement for Aminnia

Conflict of Interest Statement for Hosseini-Monfared

Conflict of Interest Statement for Kazemi

Conflict of Interest Statement for Pourshahryari

IRCT EN
