# Supplementary material for: Ascorbic Acid Reduces the Blood Boss After Total Knee Arthroplasty: Insights From a Randomized Controlled Trial
Source: Arthroplast Today. 2025 Feb 1;32:101618. doi: 10.1016/j.artd.2025.101618 (PMC11836488; doi:10.1016/j.artd.2025.101618)
Supplement: Conflict of Interest Statement for Poursalehian [file mmc3.docx]

# CONFLICT OF INTEREST STATEMENT

***American Association of Hip and Knee Surgeons***

(Adopted from the American Academy of Orthopaedic Surgeons disclosure statement)

The following form **must be filled out completely and submitted by each author (example, 6 authors, 6 forms).**

**All items require a response. If there is no relevant disclosure for a given item, enter "*None*.”**

Manuscript Title: Ascorbic Acid Reduces the Blood Boss After Total Knee Arthroplasty: Insights from a Randomized Controlled Trial

1. Royalties from a company or supplier (The following conflicts were disclosed) None

2. Speakers bureau/paid presentations for a company or supplier (The following conflicts were disclosed) None

3A. Paid employee for a company or supplier (The following conflicts were disclosed) None

3B. Paid consultant for a company or supplier (The following conflicts were disclosed) None

3C. Unpaid consultants for a company or supplier (The following conflicts were disclosed) None

4. Stock or stock options in a company or supplier (The following conflicts were disclosed) None

5. Research support from a company or supplier as a Principal Investigator (The following conflicts were disclosed) None

6. Other financial or material support from a company or supplier (The following conflicts were disclosed) None

7. Royalties, financial or material support from publishers (The following conflicts were disclosed) None

8. Medical/Orthopaedic publications editorial/governing board (The following conflicts were disclosed) None

9. Board member/committee appointments for a society (The following conflicts were disclosed) None

**Each author must sign AND print or type his/her name, date and submit a separate form**

In addition, one BLINDED Conflict of Interest form (no author names used) should be submitted per manuscript with all author disclosures.

Mohammad Poursalehian 6/28/2024
