# Supplementary material for: Ascorbic Acid Reduces the Blood Boss After Total Knee Arthroplasty: Insights From a Randomized Controlled Trial
Source: Arthroplast Today. 2025 Feb 1;32:101618. doi: 10.1016/j.artd.2025.101618 (PMC11836488; doi:10.1016/j.artd.2025.101618)
Supplement: IRCT EN [file mmc8.pdf]

Evaluation the Effect of Ascorbic Acid compared to normal saline on the hidden blood loss in total knee arthroplasty

More options

Protocol summary

**Study aim**	In this study we are aiming to investigate the effect of the antioxidant agent including ascorbic acid on the amount of hidden blood loss in total knee arthroplasty (TKA).
**Design**	A double-blind randomized controlled trial with two parallel groups
**Settings and conduct**	In this randomized controlled clinical trial, 100 patients which are scheduled for TKA will be consecutively assigned into two groups in a 1:1 ratio. The patients in group A will receive three doses of ascorbic acid. The patients in group B will receive only normal saline as a control group. In this double-blind study, patients and data collectors are blinded and unaware of the patient's assigned group. Informed consent will be obtained from all the patients prior to the surgery.
**Participants/Inclusion and exclusion criteria**	Inclusion criterion: patients who are scheduled for total knee arthroplasty. Non-inclusion criteria: history of thromboembolic events like deep vein thrombosis or pulmonary embolism; a history of cardiovascular disease; clotting disorders including abnormal PT, PTT or INR; being in pregnancy or in the lactation period; drug abusers or alcoholics; severe renal dysfunction; severe infection; the preoperative hemoglobin <10 g/dL; diagnosis of inflammatory arthritis
**Intervention groups**	Intervention group: patients in this group will receive the first ascorbic acid dose (1 g in 10 mL) at the beginning of the surgery. Another dose of ascorbic acid (1 g in 10 mL) will be infused during the surgery. After the surgery, an additional dose of ascorbic acid (1 g in 10 mL) will be infused during the first 12 hour postoperative period. Control group: The patients in this group will receive only normal saline.

**Main outcome variables**	Total blood loss

General information

**Reason for update**	
**Acronym**	
**IRCT registration information**	IRCT registration number: **IRCT20221104056393N1**<br>Registration date: **2023-06-27, 1402/04/06**<br>Registration timing: **registered_while_recruiting**<br><br>Last update: **2023-06-27, 1402/04/06**<br>Update count: **0**
**Registration date**	2023-06-27, 1402/04/06
**Registrant information**	**Name**<br>Pooya Hosseini-Monfared<br>**Name of organization / entity**<br>**Country**<br>Iran (Islamic Republic of)<br>**Phone**<br>+98 21 2200 1072<br>**Email address**<br>pomonfared@sbmu.ac.ir
**Recruitment status**	**Recruitment complete**
**Funding source**	
**Expected recruitment start date**	2023-06-18, 1402/03/28
**Expected recruitment end date**	2023-06-29, 1402/04/08
**Actual recruitment start date**	*empty*
**Actual recruitment end date**	*empty*
**Trial completion date**	*empty*
**Scientific title**	Evaluation the Effect of Ascorbic Acid compared to normal saline on the hidden blood loss in total knee arthroplasty

**Public title**	The Effect of Ascorbic Acid on the blood loss in Total Knee Arthroplasty
**Purpose**	Treatment
**Inclusion/Exclusion criteria**	**Inclusion criteria:**<br>Candidates of total knee arthroplasty patients who provide written informed consent to be included in this study<br>**Exclusion criteria:**<br>history of thromboembolic events like deep vein thrombosis or pulmonary embolism history of cardiovascular disease like myocardial infarction or atrial fibrillation being in pregnancy or in the lactation period drug abusers or alcoholics severe renal dysfunction severe infection the preoperative hemoglobin less than 10 g/dL diagnosis of inflammatory arthritis like rheumatoid arthritis, pigmented villonodular synovitis and etc clotting disorders including abnormal PT, PTT or INR
**Age**	No age limit
**Gender**	Both
**Phase**	N/A
**Groups that have been masked**	• Participant<br>• Outcome assessor
**Sample size**	Target sample size: **100**
**Randomization (investigator's opinion)**	Randomized
**Randomization description**	In this randomized controlled clinical trial, the patients will be consecutively and alternately (one in between) assigned into two groups in a 1:1 ratio.
**Blinding (investigator's opinion)**	Double blinded
**Blinding description**	In this double-blind study, patients and the data collector are blinded. Patients are kept unaware of their group assignment and the intervention they are receiving. Patients in both groups receive serums with covers so that the content will not be visible to the patients. Both the treatment and control groups receive identical instructions and support. The data collector is kept blind to the group assignment of each patient. Collecting data including laboratory values after the surgery and clinical examinations will be performed without knowing the

intervention done for the patient and only according to their records number.

**Placebo**	Not used
**Assignment**	Parallel
**Other design features**	

Secondary Ids

empty

Ethics committees

1

**Ethics committee**	**Name of ethics committee**<br>Ethics committee of Shahid Beheshti University of Medical Sciences<br>**Street address**<br>Azar dead end alley, Sharifi Manesh street., Pole Roomi, Tajrish<br>**City**<br>Tehran<br>**Province**<br>Tehran<br>**Postal code**<br>1964714953
**Approval date**	2023-06-14, 1402/03/24
**Ethics committee reference number**	IR.SBMU.RETECH.REC.1402.165

Health conditions studied

1

**Description of health condition studied**	Total Knee Arthroplasty
**ICD-10 code**	M17.9
**ICD-10 code description**	Osteoarthritis of knee, unspecified

Primary outcomes

1

**Description**	total blood loss
**Timepoint**	Before intervention, 24h after intervention
**Method of measurement**	amount of Hemoglobin drop

Secondary outcomes

1

**Description**	Transfusion rate
**Timepoint**	Before the intervention, 24h after the intervention
**Method of measurement**	number of units of blood transfused

Intervention groups

1

**Description**	Control group: The patients in the control group will receive only normal saline.
**Category**	Treatment - Surgery

2

**Description**	Intervention group: The patients in the intervention group will receive the first ascorbic acid dose (1 g in 10 mL) at the beginning of the surgery. Another dose of ascorbic acid (1 g in 10 mL) will be infused during the surgery. After the surgery, an additional dose of ascorbic acid (1 g in 10 mL) will be infused postoperatively during the first 12h.
**Category**	Treatment - Surgery

Recruitment centers

1

Recruitment center

Name of recruitment center
Akhtar Hospital

Full name of responsible person
Seyed Morteza Kazemi

Street address

Azar dead end alley, Sharifi Manesh St., Pole Roomi, Tajrish

City
Tehran

Province
Tehran

Postal code
1964714953

Phone
+98 21 2200 1072

Email
pomonfared@gmail.com

Sponsors / Funding sources

1

Sponsor

Name of organization / entity
Shahid Beheshti University of Medical Sciences

Full name of responsible person
Dr Afshin Zarghi

Street address
Azar dead end alley, Sharifi Manesh St., Pole Roomi, Tajrish

City
Tehran

Province
Tehran

Postal code
1964714953

Phone
+98 21 2200 1072

Email
boneresearch@sbmu.ac.ir

Grant name

Grant code / Reference number

Is the source of funding the same sponsor organization/entity?
Yes

**Title of funding source**	Shahid Beheshti University of Medical Sciences
**Proportion provided by this source**	100
**Public or private sector**	Public
**Domestic or foreign origin**	Domestic
**Category of foreign source of funding**	*empty*
**Country of origin**	
**Type of organization providing the funding**	Academic

Person responsible for general inquiries

Contact

Name of organization / entity
Shahid Beheshti University of Medical Sciences

Full name of responsible person
Seyed Morteza Kazemi

Position
Professor

Latest degree
Specialist

Other areas of specialty/work
Orthopedics

Street address
Azar dead end alley , Sharifi Manesh street, Pole Roomi, Tajrish

City
Tehran

Province
Tehran

Postal code
1964714953

Phone
+98 21 2200 1072

Email
kazemimort@gmail.com

[illegible]

Person responsible for scientific inquiries

Contact

Name of organization / entity
Shahid Beheshti University of Medical Sciences

Full name of responsible person
Seyed Morteza Kazemi

Position
Professor

Latest degree
Specialist

Other areas of specialty/work
Orthopedics

Street address
Azar dead end alley , Sharifi Manesh street, Pole Roomi, Tajrish

City
Tehran

Province
Tehran

Postal code
1964714953

Phone
+98 21 2200 1072

Email
kazemimort@gmail.com

Person responsible for updating data

Contact

Name of organization / entity
Shahid Beheshti University of Medical Sciences

Full name of responsible person
Pooya Hosseini-Monfared

Position
Medical Intern

Latest degree
A Level or less

Other areas of specialty/work
Orthopedics

Street address
Azar dead end alley , Sharifi Manesh street, Pole Roomi, Tajrish

City
Tehran

Province
Tehran

Postal code
1964714953

Phone
+98 21 2200 1072

Email
pomonfared@gmail.com

Sharing plan

**Deidentified Individual Participant Data Set (IPD)**	Yes - There is a plan to make this available
**Study Protocol**	Undecided - It is not yet known if there will be a plan to make this available
**Statistical Analysis Plan**	Undecided - It is not yet known if there will be a plan to make this available
**Informed Consent Form**	Undecided - It is not yet known if there will be a plan to make this available
**Clinical Study Report**	Yes - There is a plan to make this available
**Analytic Code**	Undecided - It is not yet known if there will be a plan to make this available
**Data Dictionary**	Not applicable
**Title and more details about the data/document**	Information about the main outcome can be shared after making the individuals undetectable.
**When the data will become available and for how long**	Access will be granted 6 months after the results of the study are published.
**To whom data/document is available**	Researchers and medical specialist
**Under which criteria data/document could be used**	based on the Ministry of Health and Medical Education criteria for clinical trials
**From where data/document is obtainable**	Orthopedic department of Shahid Beheshti Medical University

**What processes are involved for a request to access data/document**	The data will be provided after reviewing the researcher's request and providing sufficient documentation of their research and the reason for using the data.
**Comments**	

Tel:
Working hours:
8:00 - 15:30 Tehran time
11:30 - 19:00 GMT

0098 21 8670 5503

During COVID-19 Epidemic at working times:
0098 936 770 7834

Fax:
0098 21 8670 5503

Email:
irct@behdasht.gov.ir (mailto:admin@irct@behdasht.gov.ir)

Directly contacting the manager:
0098 912 778 2686

Address:
IRCT administration team,
Central Library Building, Iran University Campus,
Hemmat freeway, next to Milad tower,
Tehran, 14496-14535
Iran

Copyright © IRCT 2008-2024. All Rights Reserved.
